# General insights on obstacles to dog vaccination in Chad on community and institutional level

**DOI:** 10.3389/fvets.2022.866755

**Published:** 2022-10-12

**Authors:** Nodjimbadem Mbaipago, Alladoumngar Madjadinan, Djedou Martin Amalaman, Prisca Andrée Ndour, Jakob Zinsstag, Kathrin Heitz-Tokpa, Monique Lechenne

**Affiliations:** ^1^Centre de Support en Santé Internationale, N'Djamena, Chad; ^2^Université Peleforo Gon Coulibaly, Korhogo, Côte d'Ivoire; ^3^École Inter-États des Sciences et Médecine Vétérinaires, Dakar, Senegal; ^4^Swiss Tropical and Public Health Institute, Basel, Switzerland; ^5^University of Basel, Basel, Switzerland; ^6^Centre Suisse de Recherches Scientifiques en Côte d'Ivoire, Abidjan, Côte d'Ivoire

**Keywords:** rabies, cultural context, dog vaccination, veterinary services, Chad

## Abstract

Domestic dogs are responsible for 95% of all human rabies cases worldwide and continue to be the main reservoir for this fatal virus in African and Asian countries. Interrupting the spread of the disease in the domestic dog population is therefore necessary for long-term, sustainable rabies control. Chad has been recognized as a rabies-endemic country since 1961, but no national control strategy is in place to date and dog vaccination coverage is very low. This qualitative, descriptive study aims to describe the main barriers to dog vaccination on both the community and the institutional level from a socio-anthropological point of view in Chad. The study was embedded in an overall project conducted from 2016 to 2018, to determine rabies burden and vaccine demand in West and Central Africa, funded by GAVI, the vaccine alliance. Data collection was conducted on the occasion of the project's closing workshops with stakeholders organized between August to September 2018 in the four (4) project areas: Logone Occidental, Ouaddaï, Hadjer Lamis and Chari Baguirmi. We conducted interviews and focus group discussions (FGD) among veterinary officers and dog owners. Participants were selected purposively based on their place of residence (dog owners) or work place (veterinary officers) and their previous contact with the project through reporting (dog owner) or management (veterinary officers) of a suspect dog rabies case. In each region, one FGD was organized with dog owners, and one FGD with heads of veterinary posts. At the end of the FGDs, a few participants were randomly selected for interviews. In addition, in each region an interview was conducted with the head of the livestock sector, the chief district medical officers and the head of a civil society association. The identified barriers to dog vaccination access are grouped into three main aspects: the economic, the socio-cultural and the institutional level. Economic constraints encountered relate to the cost of the vaccine itself and the expenses for transporting the dogs to the vaccination site. The cultural belief that the vaccine will have an impact on the therapeutic properties of dog meat for consumers (observed in Southern Chad), and the fact that dogs are considered impure animals in Muslim faith, which prohibits handling of dogs, are obstacles identified on the sociocultural level. At the institutional level, the unavailability of vaccines in veterinary services, the lack of communication about the law on dog vaccination, the absence of rabies in the training curricula of veterinary agents, and the lack of intersectoral collaboration limit vaccination coverage. In order to improve vaccination coverage and rabies surveillance with a view to eradicate rabies by 2030, communication strategies that are adapted to the context and that take cultural obstacles into account must be put in place in a synergy of interdisciplinary action. In addition, factors such as affordability, geographical access and availability of dog rabies vaccines needs to be addressed throughout the country. Although our study design did not allow a detailed analysis of obstacles related to socio-economic level, gender and age the broad insights gained can provide general guidance for future interventions in Chad and similar countries.

## Introduction

Rabies, a lethal zoonotic disease, is mostly transmitted to humans by infected dogs and mainly affects marginalized communities in Africa and Asia (1). In many low-income countries, the classical rabies virus (RABV) is endemically present in dog populations (2, 3). At the same time most of the dogs are free roaming leading to frequent human to dog contact (4). Humans usually get infected accidentally through a bite, scratch, or lick of excoriated skin by a rabid animal and tragically, children are disproportionally affected (1). The disease is almost invariably fatal once clinical signs appear (5). Timely administration of rabies vaccine to bite victims can prevent the onset of rabies (6), but this measure called Post-Exposure-Prophylaxis (PEP) is very costly, hard to access for marginalized communities and most importantly does not reduce overall exposure risk for those communities. With 25,000 deaths per year, Africa is one of the continents most affected by rabies (7). The recognized most cost-effective control measure that also holds the potential for elimination of dog-mediated human rabies is large-scale vaccination of dogs (8–10). Rabies control became a flagship of the One Health approach because it illustrates very well the added value of intervention in animals to improve health not only in animals but for humans and the ecosystem overall (11), which is the core idea behind this concept. The feasibility of dog vaccination to reduce the burden of human rabies is proven by the success of this intervention to eliminate canine rabies from large parts of Latin America (12) and by many successful local interventions in Africa and Asia (13–15). However, the same studies point out the need for sustained control measures, the necessity for locally adapted methods and the importance of accurate sensitization through local awareness campaigns. For example in the case of N'Djamena, the capital city of Chad, two consecutive mass vaccination campaigns were sufficient to temporarily eliminate rabies from the city, but after the interventions, rabies was reintroduced from the rural area (16). During the same vaccination intervention, considerable differences were noted between the achieved vaccination coverage in different quarters of the town depending on the socioeconomic and cultural context (13). In a similar intervention in Bamako, Mali, vaccination coverage did not achieve the level needed to interrupt rabies transmission in the dog population (17). This indicates that sociocultural factors determine the accessibility of dogs to vaccination because they determine adequacy and acceptance of the intervention measures, as described by Obrist et al. (18). Local economic and socio-cultural context also determines the most effective tools and strategies for awareness raising. Therefore, it is important to study human attitudes and practices toward dogs and rabies in order to better plan and undertake dog vaccination campaigns.

To achieve sustainability, dog vaccination campaigns need to be institutionalized and taken over by the governmental veterinary services. Therefore, institutional factors also influence the long-term success of vaccination interventions. Finally, accessibility of dog vaccination is heavily impacted by affordability, especially in the context of livelihood insecurity faced by many dog owners in endemic areas (19).

In Chad, as in many other low-income countries, the accessibility of health facilities is often challenging which has a large impact on access to PEP (20). At the same time incidence of rabies in the dog population is observed to be high in areas where surveillance is implemented (21). Therefore, it would be all the more important to decrease exposure risk through dog vaccination. However, veterinary services are even more neglected than human health services and are mostly limited to livestock vaccination. Although vaccination of dogs in Chad is compulsory by law, the estimated vaccination coverage of 0.5% in the dog population is extremely low (22). There exists no governmental led national dog vaccination strategy. Past interventions, mainly in the capital N'Djamena have been based on intervention research projects implementing dog mass-vaccination for a short period of time and in a limited geographical setting.

Dog keeping households are widespread in Chad, and dogs are used for many purposes, ranging from protection to hunting and breeding (22). Dog ownership is most common in the predominantly Christian southern regions of the country, but is also observed in Muslim areas (22). According to Anyiam et al. (23), Chad's dog population is estimated at 1,205,361 dogs. Only around 10% of the overall dog population in Chad are stray (ownerless) dogs (16), but most of the owned dogs roam freely due to lack of means to adequately feed the animals. Dogs supplement their diet from food scraps in the environment such as human feces, wildlife, slaughter offal, and other waste. This creates a lot of contact with people, especially children, who are often unsupervised. Challenges are noted on many levels and show that rabies is a topic that deserves special attention. This paper aims to contribute to the knowledge basis for future planning of control programs in Chad through identification of the socio-economic, socio-cultural and institutional barriers that limit access to dog vaccination in the various cultural contexts of the country. These are very diverse and range from regions dominated by sedentary farmers with a Christian background to regions with a predominantly pastoralist population and a mainly Muslim rooted culture.

## Materials and methods

### Study background

**Figure 1:**
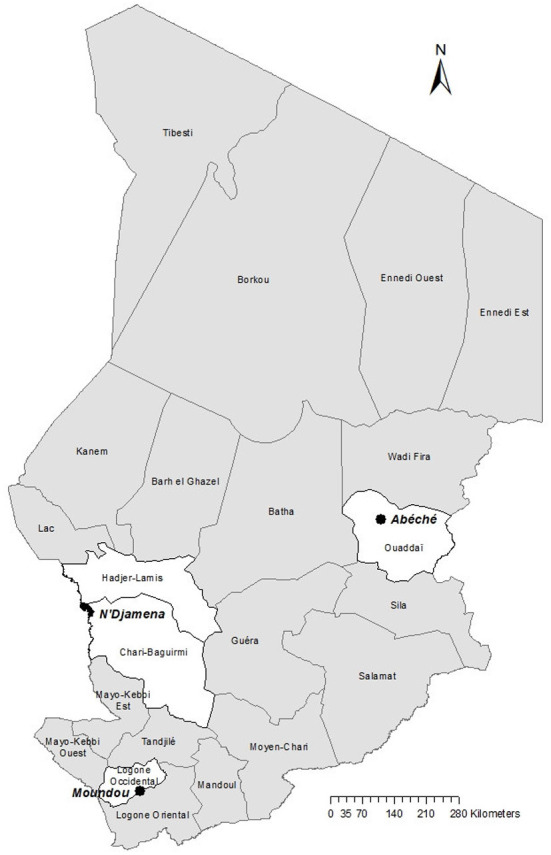
Map of Chad showing the provinces in which the GAVI-project was implemented.

The qualitative study presented in this paper is part of a large-scale research project implemented in Chad, Côte d'Ivoire and Mali, to estimate the burden of rabies and vaccine demand in West and Central Africa, funded by GAVI, the vaccine alliance (GAVI-project) (24). In Chad, this research project lasted from January 2016 to September 2018 and was implemented in three study areas belonging to four administrative provinces (Figure 1). The provinces were selected based on their various demographic and socio-cultural characteristics to cover the range of backgrounds in the country. The quantitative studies undertaken during the project are already published in different papers. They included a household survey on bite occurrence (20), a health facility-based study on reporting of bite cases, PEP access and follow-up (20) and extension of animal surveillance to the project areas (21).

The quantitative studies combined revealed a high occurrence of dog rabies in Chad and in consequence, a high dog to human transmission risk that calls for urgent control measures such as dog vaccination. At the same time, the study team members encountered differences in attitudes toward dogs and practices for rabies prevention across the study areas that might hamper future control interventions, in particular dog vaccination. Therefore, we identified the need to investigate on these differences through a qualitative study, which for the first time, would provide some broad insights on the influence of socio-cultural factors on rabies control in Chad. Data collection for the study was conducted during the closing workshops of the project conducted for each study area. The study's aim is to gain a general overview of obstacles to dog vaccination against rabies in Chad combining the perspectives of different cultural and socio-economic contexts on the population/client side with institutional perspectives of the dog vaccination service provider side (in our case governmental veterinary services). It is a socio-anthropological study, based on a qualitative approach with focus group discussions (FGD) and individual semi-structured interviews (25). Such a research design allows gaining real life examples of how dogs are perceived and treated, the practices the population and animal health professionals adopt regarding dogs in the context of rabies prevention as well as the underlying institutional factors influencing these practices. A detailed analysis of different socio-demographic characteristics, gendered analysis at household level or a deeper investigation into the dynamics at play in government institutions were beyond the scope of this study.

### Description of study areas

The first study area includes the province Logone Occidentale, with the city of Moundou, the economic capital and second largest town of Chad (Figure 1). Logone Occidentale is the smallest province in southwestern Chad and is populated mostly by Christians and animist communities. Outside of the regional capital in the rural areas, people live mostly on small-scale farming. The population of the Logone Occidentale province is estimated at 790,694 inhabitants with a human density of 88.68 inhabitants per square kilometer (26). The second zone covers the sparsely populated Ouaddaï region in the northeast, with a predominantly Muslim population. Its regional capital is Abéché situated very close to the Sudanese border in the northeast of Chad (Figure 1). In 2009, Ouaddaï had a population of over 700,000 with a density of 24 inhabitants per square kilometer (26). In the vast rural areas of this province, people are mainly pastoralists. The third study area is defined by a 100-kilometer radius around the capital city of N'Djamena situated in the central-west of the country. It includes parts of the province Chari Baguirmi to the south of the capital, and parts of the province Hadjer Lamis to the north of the capital (Figure 1). These two administrative provinces have a culturally mixed population of sedentary farmers and pastoralists. The population density is about 18 inhabitants per square kilometer (26).

The study areas were broadly divided into urban and rural strata: The provincial capitals (Moundou and Abéché) are considered urban and the rest of the respective province is considered rural. The study area covering parts of Chari Baguirmi and Hadjer Lamis was considered rural.

### Target population, recruitment, and participants' background

Based on the experience form the various quantitative studies of the GAVI project, we identified the following stakeholder groups to be interviewed:

Community level:
- Dog owners
- Heads of the regional civil society association

Institutional level
- veterinary officers (heads of veterinary posts)
- administrative authorities of the study regions (heads of district veterinary delegations, heads of regional livestock sectors and chief medical district officers).

For the purpose of the study a dog owner is defined as a person that keeps one or more dogs at his household for which he provides care to some extent (food, shelter etc.) and to which he attributes a role (watchdog, guard dog, etc.). Dog owners were selected in a purposive manner with the help of veterinary officers. Most of them had no formal employment and were farmers, herders, informal traders, housewives or students. The only selection criteria for the institutional representatives was the prior participation in the extended animal surveillance and bite reporting activities of the overall GAVI-project. Public veterinary officers in Chad most often do not have a university degree in veterinary science, but are graduated livestock technicians. Veterinary officers and dog owners came from various districts in rural and urban settings of the respective regions. In total, the study gathered 42 participants in 6 different FGDs and conducted 21 individual interviews. The discussion points of the FGDs and the interview guides are provided in the Supplementary Document (S1). Two thirds of the institutional representatives were Muslims (18) and the other third Christians (9). Study participants representing the population and dog owners included 13 Christians and 11 Muslim. It is important to note that local belief systems based on a rich ethnic background persist in the population, even if people go to church or pray at the mosque. Furthermore, formal education with a Francophone heritage add to the cultural complexity. As mentioned above the aim of the study was not to analyze the different socio-cultural profiles in detail, but to identify major trends about beliefs and practices concerning dogs, their vaccination, rabies and institutional shortcomings. Considering the rather broad level of analysis that we aimed at (rather than depth), we did not pay particular attention to gender, neither age (see Table 1 below). However, both demographic characteristics are mentioned to provide some information to the reader about the positionality of the different quotes.

**Table 1:** Socio-demographic characteristics of research participants.

**Data collection tool**	**Site**	**Sex**	**Age range** **(years)**	**Religious affiliation**	**Dog owners**	**Heads of civil** **society** **associations**	**Heads of veterinary** **posts and livestock** **delegation**	**Heads of** **livestock** **sectors**	**Chief district** **medical** **officers**
FDG	Logone occidental	Men: 5 Women: 2	22–67	Christian: 6 Muslim: 1	7	-	-	-	-
		Men: 6 Women: 1	33–59	Christian: 5 Muslim: 2	-	-	7		
FDG	Ouaddaï	Men: 6 Women: 1	25–58	Muslim: 7	7	-	-	-	-
		Men : 6 Women: 1	34–50	Muslim: 6 Christian: 1	-	-	7	-	-
FDG	Hadjer Lamis and Chari Baguirmi	Men: 5 Women: 2	30–66	Christian: 3 Muslim: 4	7	-	-	-	-
		Men: 4 Women: 3	44–52	Christian: 4 Muslim: 3	-	-	7	-	-
Interview	Logone Occidental	Men: 5 Women: 2	43–65	Christian: 6 Muslim: 1	3	1	1	1	1
Interview	Ouaddai	Men: 6 Women: 1	40–56	Muslim: 7	3	1	1	1	1
Interview	Hadjer Lamis and Chari Baguirmi	Men: 5 Women: 2	45–57	Christian: 2 Muslim: 5	3	1	1	1	1

Source: field data.

Three (3) languages were used depending on the region and characteristics of the interviewees to allow everyone to participate in the discussion. French was used to talk to service providers in all regions. To talk to dog owners in the Logone Occidental region in southern Chad, Ngambay was used, whereas in rural N'Djamena and the Ouaddaï region, Arabic was used in interviews and FGDs.

In a society that values seniority, the constitutions of groups in relation to age always has an influence on how participants of FGD express themselves. Therefore, individual interviews were added to complement the data sources (triangulation). Men and women were mixed in FGDs of dog owners. This choice was taken based on the focus on economic, religio-social and institutional factors (rather than gender). For the client vs. provider perspective adopted in this article, it was crucial not to mix service providers with the client (dog owner) side, as this may have limited dog owners from expressing their views on the service received out of fear of disadvantages in case of future consultations. Before the discussions and interviews, each participant signed an informed written consent.

Table 1 describes the number of FGD and interviews by study site and the study participants' backgrounds.

### Data collection techniques and tools

This qualitative data collection was conducted on the occasion of the above mentioned stakeholder workshop, during which the results of the quantitative studies undertaken during the GAVI-project were shared. The workshops were held between August to September 2018 distributed as follows: from August 22 to 23, 2018 in Abéché, from August 27 to 28, 2018 in Moundou and on September 10, 2018 in N'Djamena.

Data were collected by FGDs and by direct semi-structured interviews using an interview guide (S1). With dog owners, veterinary officers and heads of livestock delegations both in-depth interviews and FGDs were conducted. With heads of civil society associations, district medical officers and heads of livestock sectors only individual interviews were conducted. The FGDs were held separately for community representatives and the institutional representatives (see Table 1). This allows to juxtapose perspectives on dog vaccination from the client's point of view (the dog owners) with the institutional point of view, as well as to compare formal rules with actual vaccination practices. There were seven participants per FGD. In average, the FGD lasted about 45 min.

The interview guide was structured along the following thematic aspects: Economic and socio-cultural factors affecting access to dog vaccination and institutional factors that hinder access to dog vaccination. The interviews lasted about 30 minutes each.

### Data processing and analysis

With the participants from the institutional level, interviews were conducted in French. With the dog owners, Arabic and Sara was used, in addition to French. The lead author who did the field research speaks all three languages fluently. The data collected through the FGDs and individual interviews were translated into French if needed and transcribed and entered into Word version 2010 and then processed using MAXQDA version 2018 software. The data was analyzed using content analysis. Coding was done inductively. The text broken down into thematic paragraphs and coded with the help of the software.

The first and second author of this paper were members of the local GAVI-project team and as such involved on all levels of the quantitative data collection: Household survey, bite case reporting and animal rabies case surveillance. In addition, the main author was responsible for the free hotline established during the project to facilitate communication between the animal health and human health workers and reporting of bites or animal rabies incidents by the public (27). Together with the main author's participation in prior rabies research studies in Chad (22), the presence in the field and close contact with both the community and the institutions during the GAVI-project period has allowed him to gain the needed experience to appropriately interpret the FGDs and interviews.

### Ethical considerations

Ethical approval for the overall study, covering research activities in the three GAVI-project countries (Mali, Côte d'Ivoire and Chad), was granted by the Ethics Committee of Northern and Central Switzerland (EKNZ). The specific research activities of the GAVI-project in Chad received research approval from the Chadian Ministry of Public Health (MSP) (No. 1569/MSP/SE/SG/DGAS/2016) and the National Bioethics Committee of the Chadian Ministry of Higher Education (No. 298/PR/MESRS/SG/CNBT/2016). Written consent was obtained from each participant before data collection.

## Results

The data presented here address the obstacles to dog vaccination in Logone Occidentale (LO) province, in Ouaddai (O) province and rural Ndjamena (Chari Baguirmi (CB) and Hadjer-Lamis (HL) provinces) of Chad. It is structured in two sections: The first section presents the economic and sociocultural factors that constitute obstacles to dog vaccination on the population side, and the second point deals with the institutional factors that prevent dogs from being vaccinated on the provider/veterinary side. At the end of the result section, we summarize the topics discussed in a table to give a general overview (Table 2).

**Table 2:** Summary of factors influencing access to dog vaccination in Chad.

**Dimension**	**Variables**	**Obstacles**	**Propositions**
Economic	Cost of the vaccine	- Low purchasing power - Transportation costs	- Provide vaccine free of charge - Increase local availability of vaccine
	Geographic accessibility	- Distance to vaccination site	- Conduct door-to-door vaccination
	Means for transport	- Transportation difficulties - Aggression by other dogs	- Conduct door-to-door vaccination
Sociocultural	Social function of the dog	-Belief in the negative effects of the vaccine on dogs	-Conduct awareness campaigns
	Perception of the dog	- Negative attitude toward the dog as an impure animal - Lack of awareness	-Conduct awareness campaigns
Institutional	Vaccine availability in veterinary posts	- Lack of electricity - Supply difficulties	-Use of solar panels for conservations -Improve supply management
	Reinforcement of the law on dog animal vaccination	- Lack of data on rabiess - Low involvement of authorities	- Increase surveillances - Conduct participatory workshops on rabiess - Conduct awareness campaigns
	Knowledge of the animal and human health workers about rabies	- Absence of the rabies topic in the curricula	- Strengthen rabies in the training of medical and veterinary studentss - Conduct joint training workshops
	Intersectoral collaboration	- Lack of communication - Lack of notification of rabies cases - Neglect of rabies on the national level	- Improve intersectoral communication on rabiess - Increase reporting of rabiess - Allocate means for One Health collaboration

Source: field data.

### Economic and socio-cultural factors affecting access to dog vaccination

Economic and sociocultural factors that hinder dog vaccination include: (1) the high cost of the vaccine; (2) the distance between home and veterinary posts; (3) the lacking means of transporting the dog to the vaccination post and (4) the use of dog meat for therapeutic purposes in some socio-cultural milieus.

#### Cost of the vaccine

The cost of the animal rabies vaccine varies between 10,000 and 15,000 CFA francs (15 to 23 Euros) depending on the locality in Chad. The majority of respondents found the price of the vaccine very expensive. With a Gross Domestic Product (GDP) of less than one (1) Euro (<500 CFA francs) per day (https://www.data.worldbank.org/country/chad), most people were unable to pay for vaccines. Respondents from all focus groups in the different regions said that the high cost and local unavailability of the vaccine prevent them from vaccinating their dogs.

“*Dog vaccines are too expensive, and it is difficult to find them even in Beinamar. I went but I could not find it.”* (Dog owner, Christian, M, 31, LO)

“*When I wanted to vaccinate my dog, I looked for the vaccine until Koumra [town about 275 km from owner's place of residence] and they charged me 80.000 FCFA [122 Euro], I was unable to buy it.”* (Dog owner, Christian, M, 46, LO)

“*The vaccine is too expensive, that's why we don't vaccinate the dogs. The state must make the vaccine free for us, otherwise we cannot vaccinate our dogs.”* (Dog owner, Muslim, M, 57, CB)

“*The state must subsidize the price of vaccines to allow the population to get them.”* (Dog owner, Muslim, M, 53, HL)

Some study participants even say:

“*We don't have money to pay for bread and you ask us to pay for vaccine that is expensive for dog only.”* (Dog owner, Christian, W, 41, CB)

“*The vaccine costs are too much, the state must give it for free. We want to vaccinate our dogs well, but with this price, we can't.”* (Dog owner, Christian, M, 32, LO)

“*In our community, dogs are not vaccinated, because of the cost of vaccines. A dose of dog vaccine costs up to 20,000FCFA [30 Euro]. And our purchasing power does not allow us to buy them*.” (Dog owner, Muslim, M, 48, O)

These comments show that there is a willingness to vaccinate dogs in the different communities, but the cost of the vaccine is a common barrier to access on the national scale. Furthermore, dog owners complain about the unavailability of vaccines at institutional level and lack of information where to receive it:

“*One day, I took my dog to the veterinary station in Beinamar. But the agents told me that they do not have a refrigerator to store the vaccines*. (Dog owner, Christian, W, 39, LO)

“*We are not informed about where dog vaccines are sold.”* (Dog owner, Christian, M, 27, LO)

#### Geographical accessibility of the vaccine

The distance between homes and veterinary facilities that might provide vaccine is a major concern for our study participants. In all four regions of the study, rabies vaccines are stored in the regional capitals (Moundou, Abéché, Massakory, and Massenya), because of the lack of electricity for storage at veterinary district levels. In Logone Occidental, the distance between the district veterinary office and the provincial livestock delegation that manages the vaccines is between 60 and 80 km. In Ouaddaï, it is between 150 and 200 km. The one of Chari-Baguirmi is in the range of 80–100 km away and in Hadjer Lamis, the provincial delegation in Massakory is located between 100 and 150 km from the district offices of the region. Thus, when in need, people are obliged to travel at least 60 km to have their animals vaccinated and some up to 200 km. The average cost of a single trip for transportation between the districts and the regional livestock delegation is 3,000 to 6,000 FCFA *[4.5 to 9.- Euro]*. Given the poor condition of the road, people and animal have to calculate about 5–8 h (in the rainy season even up to 10–12 h) to get the vaccine. Those who do not have a means of transportation find it impossible to have their dogs vaccinated. Interview respondents said:

“*It must be recognized that the rabies vaccines are only available in Moundou. And we who are in the other localities, do not have the possibility to keep them because we do not have the electricity to keep them. When someone wants to vaccinate their dog, they are directed to Moundou and because of the distance, they refuse to go.”* (Veterinary officer, Christian, M, 58, LO)

“*We have problems with vaccine supply. When it rains, the road is cut by rainwater and we are isolated. It takes 3 to 4 months for our roads to be cleared. Under these conditions, access to vaccine is difficult”* (Veterinary officer, Muslim, M, 46, HL)

*Our district is very far from the regional hospital in Abéché and sometimes in Abéché itself, we cannot find vaccine for the dogs. If needed, we are sent all the way to Ndjamena, more than 1,000 kilometers to get the vaccines.”* ( Dog owner, Muslim, M, 39, O)

During the focus groups, respondents expressed themselves in these terms:

“*There are no vaccines in the veterinary station at our home in Abdi. If someone wants to vaccinate their dog, we send them to Abeche. But as the journey is long, the owners of the dogs refuse to go. We have no way to store the vaccines here.”* (Dog owner, Muslim, M, 53, O)

“*We can't transport the dogs more than 150 kilometers for vaccination. One has to send the vaccines to the districts.”* (Dog owner, Muslim, M 60, O)

“*During the rainy season, with the rising waters, we are cut off from other towns for at least 3 months. It is difficult for us to get supplies of vaccine.”* (Veterinary Officer, Christian, M, 47, O)

“*Everything is centralized in Abéché. We need to supply the other districts with vaccine to reduce the distance and facilitate accessibility.”* (Veterinary Officer, Muslim, M, 50, O)

“*In the rainy season, we have difficulties accessing products such as vaccine because the conservation conditions are not met. To go to Ndjamena, the roads are impassable because of the rainwater.”* ( Veterinary Officer, Muslim, 36, O)

“*To get the vaccine, we have to travel several kilometers because the vaccine does not exist in our district. So this distance does not allow us to access the product.”* (Dog owner, Christian, 42, CB).

“*To get your dog vaccinated, you have to go all the way to Ndjamena, because here in Dourbali (106 km de Ndjamena), there is no rabies vaccine.”* (Dog owner, Christian, M, 52, CB)

#### Means of transporting dogs to the vaccination post

One of the difficulties raised by our respondents is the lack of means to transport the dog to the vaccination site. In this regard, about three quarters of the respondents stated that it is impossible to drag the dog on foot for miles to vaccinate it. Since most of the dogs are free roaming taking the dog on foot for vaccination entail the risks for dog-to-dog aggression. In addition, there is the risk that the dog might aggress other people in the street. The cost of transporting dogs in a public vehicle (between 2,000 and 3,000 CFA francs, or 3 to 5 Euros) makes it difficult to travel longer distances to access the vaccine. Furthermore, in the case of public transport, some truck owners refuse to transport dogs, because according to their belief the dog is an animal of misfortune whose presence in the truck can cause accidents.

Some of the comments made in the interview were:

“*When you bring your dog somewhere, the other dogs chase you and the dogs fight.”* (Dog owner, Christian, M, 54, HL)

“*The dog is a complicated animal. If you take it somewhere, the other dogs will follow you and fight, or even bite you. If you take him in the truck, he attacks the other passengers. That's why we can't get our dogs vaccinated.”* (Dog owner and head of social civil society association, Christian, M, 31, LO)

“*I would like to vaccinate my dog, but I do not know how to transport it to the vaccination site. If the vaccine is in our district, we will get our dogs vaccinated. But we have to go all the way to Moundou. We don't know how to transport our dogs*.” (Dog owner, Muslim, W, 39, HL)

“*Transporting dogs to the vaccination site is always a problem in our communities. We need to organize door-to-door vaccination. We don't know how to transport the dogs to the vaccination [site]*.” (Dog owner, Muslim, M, 46, O)

“*We have no means of transporting the dogs to Ndjamena for vaccination. And the vehicle owners refuse to take the dogs, because they will cause an accident.”* (Dog owner, Muslim, M, 60, CB)

#### Social value and perception of the dog

In addition to its value as a guardian and pet, many male respondents of the Logone Occidental, region of southern Chad mentioned that they eat dogs due to its ascribed therapeutic properties. This cultural practice of dog meat consumption persists in Christian communities in southern Chad and is not practiced among Muslims. Several research participants mentioned that the vaccination of dogs could destroy the virtues contained in the dog. The statements about this specific function and perception of the dog are as follows:

“*The meat of the dog is a medicine for us. It preserves against bad spells, evil spirits and rejuvenates the cells of the body.”* (Dog owner, Christian, M, 59, LO)

“*We raise the dog to eat. When you hear the dog howling at night like a wolf, it means it has seen a spirit. So we eat the dog's meat to protect ourselves from evil spirits. But when you vaccinate the dog, it loses all these virtues and it has no longer any effect.”* (Dog owner, youth Association Leader, Christian, M, 43, LO).

“*The dog is used for hunting in our country. But when it dies, we make medicine with its flesh against disease. That's why we don't want to vaccinate it.”* (Dog owner, Christian, M, 39, CB)

To the contrary, research participants with a predominantly Muslim background in the Ouaddaï region, consider the dog as an impure animal that should not be approached according to the religious and cultural practices. According to our study participants, even if a dog owner is willing to handle the dog and bring it to a vaccination location, the community's negative perception of someone handling a dog is an obstacle for owners to bring their dogs to vaccination.

“*The dog is not appreciated in our community. Those who approach the dog are also hated. I can't take my dog to vaccination because of the community's view.”* (Dog owner, Muslim, M, 41, O).

“*At home, if you are seen handling the dog or touching the dog, people do not greet you, and do not eat with you in the same dish. You are considered dirty, unclean. We don't accept the dog near us. Even if we raise it for our own safety, it stays in the front yard and we give it food, but it does not come near the family because it is too dirty*.” (Veterinary officer, Muslim, M, 54, O)

Both the positive perception of dog meat on the one side and the negative perception of the dog as a dirty animal on the other side constitute important cultural barriers to dog vaccination.

### Institutional factors that hinder access to dog vaccination

This section focuses on institutional factors that limit access to vaccination. These include the absence of vaccines at veterinary posts, lack of communication about the Animal Health Law, lack of awareness of rabies among veterinary officers, and lack of intersectoral collaboration.

#### Unavailability of vaccines at veterinary posts

When asked if the vaccine is available at the veterinary posts, more than half of our respondents said that there is no vaccine at the posts, which confirms the experiences shared by the dog owners. In the district of Moundou, which is the vaccine storage center, cases of vaccine shortages have been noted. In this regard, the shortages are noted during certain periods as a result of lacking supply chain management, as the respondents stated during the interviews:

“*When we order the vaccine, it takes time to reach us and this can create the shortages.”* (Veterinary officer, Muslim, M, 34, O)

“*We looked for the vaccine in all the districts of the region, but we did not find it. We are told that only the district of Moundou has the vaccine. I went there but they tell me there is a break”* (Dog owner, Christian, M, 51, LO)

The absence of rabies vaccines at local veterinary posts results in the barriers to accessing dog vaccination described above such as geographical distance coupled with transport difficulties.

#### Lack of communication about the dog vaccination law

Regarding knowledge of the law that requires owners to vaccinate their pets and report cases of mandatory rabies, almost all were unaware of it.

The interviews yielded the following statements:

“*There is no law for dog vaccination in this country.”* (Dog owner, Muslim, W, 43, O)

“*Nobody told us that there is a law that obliges dog owners to vaccinate them. But when your dog bites someone and if it dies, you are arrested in prison.”* (Head of civil society association, Christian, M, 55, LO)

“*Although this law on the vaccination of dogs exists but it needs to be popularized to the public. We don't have the means to raise awareness.”* (Head of district veterinary delegation, Christian, M, 39,CB)

We note from the opinion of the respondents that the majority is not informed of the law on dog vaccination and mandatory reporting of suspected animal bites. This situation is due to the lack of communication and awareness of the population by the authorities in charge of animal health and rabies.

#### Absence of rabies in the training curricula of veterinary officers

The data collected from our respondents regarding their knowledge and practice of rabies reveal that animal health professionals do not have a good understanding of rabies. The majority of them stated that rabies was not part of their training, as some of them testified:

“*In our training, we have never heard of rabies. We do not know the manifestations or clinical symptoms of this disease in dogs.”* (Veterinary officer, Christian, M, 48, LO)

“*Rabies is considered a neglected disease by our authorities and no one cares about this disease. Yet, it kills many children. The state must develop a plan to fight this disease.”* (Head of livestock sector, Muslim, M, 57, HL)

According to them, this lack of training in rabies control is due to the negligence of the authorities in charge of animal health. The lack of knowledge of rabies by veterinary officers does not allow them to organize an awareness or communication campaign about rabies. In order to effectively communicate the danger of rabies to the population, agents must be well-trained about the subject. The absence of this training result in an access barrier related to lacking knowledge about the disease in the community as a whole.

#### Lack of intersectoral collaboration

Another important factor for access to dog vaccination is collaboration between human and animal health services. According to the data collected, the majority of our respondents stated that there is no collaboration between these two sectors:

“*We don't have collaboration as such with the human health sector. Sometimes, we meet at workshops but afterwards, each one goes its own way.”* (Veterinary officer, Muslim, M, 49, LO)

“*Since we have been practicing, we have no relationship with human health workers. Everyone evolves on their own and in case of rabies, it is difficult for us to communicate.”* (Head of livestock sector, Christian, M, 38, CB)

“*Collaboration with human health was non-existent. It is from the GAVI study that we had collaboration in the monitoring and diagnosis of the biting animal.”* (Veterinary officer, Christian, M, 43, LO)

Indeed, when there is a case of rabies, the two services must work together to adequately treat the victim, but also to follow up on the bitten animal to stop the spread of rabies. The lack of collaboration also results in inadequate notification of cases and in consequence, the burden of rabies is underestimated and authorities do not perceive the need for a national action plan.

## Discussion

The studies objective was to combine perspectives on the community and institutional level to gain a first overview of where some of the main obstacles to dog vaccination in Chad might lie. The results reveal several economic, socio-cultural and institutional factors that prevent access to dog vaccination in Chad. Some of them have been observed in other studies (8, 28), but to our knowledge this is the first qualitative study that brings together insights from both the population and the service provider level. These insights will be valuable to overcome barriers to vaccination in future planning of rabies control measures. Like for other goods, access to dog vaccination is subject to the dynamics between availability and demand. Both positive and negative factors associated to dog vaccination on the community level thus influence the factors on the institutional level and vice versa, forming an access cycle. If for example animal health professionals are not well-trained on rabies prevention and control, they are not able to provide adequate sensitization to the public to increase awareness among the community and hence demand by dog owners will remain low. On the other hand low purchasing power and limited geographical access on the community level results in low demand by dog owners and in turn, availability will remain low on the institutional level due to the unprofitability of providing dog vaccination. These examples show that the combined perspective allows us to better understand drivers or barriers influencing this access cycle.

In our study we have only looked at the provision of vaccine in public veterinary facilities, but we assume that the interplay between availability and demand will be even more negatively affect in the private veterinary sector due to the higher need for profitability. In Senegal for example the public sector funded through the government provides livestock vaccine free of charge and farmers pay only a service fee, whereas in the private distribution system, herders bear the full cost of the vaccine (29).

The study took place in four provinces of Chad that together represent the two major religio-cultural backgrounds. Moreover, the study covered the rural and urban context. The results of this study can thus provide guidance to implementation of a rabies control program at the national level. In Chadian communities, dogs are kept for a variety of reasons, ranging from protection of premises and livestock, to hunting and even consumption (22). Accordingly, the perception and role of the dog varies from one community to another based on the ethno-cultural and religious context. Despite the general picture, we are able to provide by looking at the two main religio-cultural contexts, our study has limitations. The breadth (sample size) and depth (detail) of data did not allow us to deepen the analysis of perceptions and practices that would enable us to distinguish between different ethno-linguistic groups, socio-demographic and socio-economic characteristics. Such a study could be a next step helping to design locally tailored measures to increase acceptability of interventions (18). Nonetheless, the main access barriers identified here can already be useful to prepare a general national action plan for rabies control.

A first issue to address would be the cost of the vaccine. Chad remains one of the poorest countries in the world with a Human Development Index (HDI) of 0.401 (187th out of 189 countries). The majority of study participants representing the population have low monthly incomes (<60,000 CFA francs or 90 Euros) and have no formal employment, with low purchasing power. In N'Djamena, a free mass dog vaccination campaign achieved the required level of 70% coverage (13), whereas a previous campaign in the same setting that charged around 4 USD for vaccinating a dog achieved only a coverage of 24% (30).

Studies from Uganda and Peru also show that poverty influences not only vaccination coverage, but also dog keeping practices (31, 32). Another previous study in Chad shows that lack of financial means for vaccination or to pay for PEP in a case of a bite even influences the decision to raise a dog (33). Indeed, when a rabid dog bites individuals, it is the dog owner's responsibility to take care of these victims regardless of the number of victims. Given the unavailability and cost of human rabies vaccine (20), some dog owners end up in prison, or social ties with their neighbors or the community are weakened after a dog bite incident, because they are unable to pay for PEP [main authors' own experience gained during his service as a rabies hotline agent during the GAVI-project (27)]. Risks and cost related to transport of a dog to distant veterinary facilities with vaccine in stock highlight the need for localized approaches to improve availability of services. Distance and transport were also identified to be major barriers for dog vaccination in Ethiopia (34) and Tanzania (35).

Free public rabies vaccine and bringing veterinary posts closer to communities could improve motivation of dog owners to vaccinate their animal, but our results also show that such measures alone might not be sufficient due to lack of disease awareness and socio-cultural barriers observed. A study in Ethiopia found that a dog owner's knowledge of rabies (34) is a significant predictor of the level of intention to vaccinate a dog. A finding that is supported by a study in Côte d'Ivoire reporting that low vaccine access appears to be influenced by ignorance and negligence (35). Overall these findings are not new since already back in 2010 a study conducted in several developing countries, where canine rabies is endemic, the most common reasons for dog owners not to vaccinate their dogs are lack of knowledge about the disease burden and prevention, vaccination costs, and ease of catching dogs (8). In fact, the neglect of rabies in sub-Saharan Africa is largely attributed to a lack of recognition of the infection as a significant public health threat (36).

Our qualitative data confirm the hypothesis derived from quantitative data on vaccine coverage (13) and dog population estimates (23) in Chad, that ethnic beliefs influence dog breeding/keeping in some localities. Some respondents believe that the consumption of dog meat protects them against evil spirits and helps to cure certain diseases. This power is according to them destroyed by the vaccine and therefore this aspect constitutes an obstacle to rabies control. The Christian religion considers the dog as a companion that deserves care and affection. According to Akakpo (37), a Christian priest points out that “on the day of the last judgment, all the animals rescued by your care will come to testify in your favor.”

In the Muslim communities, dogs are less appreciated. This conception is also reported in Senegal. Leye [1989, cited in Migan (38)] had stated that in Senegal, “it is common to hear that the Muslim should not raise a dog, because it is an impure animal.” Indeed, some Hadiths of Islam cited by Migan (38) teach the following: “The angel does not enter the room where there is a dog”; “The black dog is a Satan (demon)”; “If the dog licks the dish, it must be washed 6 times in natural water and the 7th time with soap.” It should be noted that in the Muslim community in Chad, the dog is called “khèleb” which means (impure, dirty, hated, smells bad) to the point where someone who is hated by society is called “wadam khèleb” which translated means “damned dog.” This concept leads the community to chase dogs off their premises and even keeps a dog, it is held in the front yard to ensure their safety, but the dogs are not allowed in the house. Such dogs that are not close to their owners and not used to be handled are difficult to reach during vaccination campaigns. In Bamako, Mali, for example, the inability to handle aggressive dogs was an important reason for non-participation in a centralized vaccination campaign (39). However, the situation is paradoxical in Pikine (a suburb of Dakar in Senegal) where the Muslim community is the one that owns most dogs (37). At a closer look the topic is more complex that commonly thought. According to cited hadiths in Migan (38), the Prophet Mohammed also said: “the best dog is the one who guards the herd and the house. He also promised paradise to the believer who quenched a dog's thirst by giving it a drink from his shoe.” In any case, in spite of religious or ethnic prohibitions, confronted with the problem of insecurity, in the cities and the countryside alike, people with different beliefs decide to keep a dog to ensure their safety.

An element that we were unfortunately not able to address with our study, is the effect of gender-dynamics on dog vaccination access. We did not particularly pay attention to gender balance during recruitment of participants and the fact that FGD were mixed certainly had an influence on the way women participated. Two new studies highlight the importance of adopting a gender sensitive approach when identifying obstacles to vaccination in the field of livestock vaccines (40). The role of managing and controlling livestock diseases in these communities was culturally ascribed to men. This is also the case for dog keeping in Chad and the decision power on whether to vaccinate or not lies almost inclusively in the hands of the male representative of the household. Moreover, dog meat consumption is limited to men (main author's experience). Even in cases where women are the primary caretakers, for example in the case of poultry farming in Senegal, there are cultural and social barriers to their ability to access vaccination services (29). Similarly, children are most often those that can handle their household dogs very well to bring to vaccination posts, but they depend on the decision of their parent. Further, more fine-grained studies of the social dynamics related to age and gender at household level would certainly provide valuable insight into gender or age related differences to access.

Obstacles also result from the low involvement of the authorities in dog vaccination campaigns. The majority of our respondents cited ignorance of the law on health police organizing dog vaccination (Law No. 04-009 2004-05-19 PR of 19 May 2004). According to this law, dog vaccination is mandatory in Chad, and sanctions are provided for all those who do not respect this law. The lack of knowledge of the law by the majority of owners is explained by the lack of awareness in the community (22, 41), lack of knowledge of animal and human health providers (42, 43) and low priorization of rabies control by concerned authorities resulting from a virtually absent rabies surveillance system (21). Results from a global study on rabies surveillance shows that Chad is not an exception in this regard (44). In fact, a very recent article reflecting on the factors hampering advances on the road to zero dog mediated human rabies cases by 2030 identified these very obstacles described here on the institutional and socio-cultural level (28). Intersectoral collaboration between human health and veterinary workers is a key factor in rabies control (11). Lack of collaboration and lack of expertise in rabies control is a challenge for the organization of vaccination campaigns. A Knowledge Attitude and Practices (KAP) study in human and veterinary health workers conducted during the GAVI-project revealed a considerable lack of knowledge about rabies and recommended treatment among the participants and confirms the negligence of the rabies topic by the training curricula of both sectors (43). The need for cross-sectoral collaboration of actors in a synergistic transdisciplinary way highlights the usefulness of a “One Health” approach for rabies control (11, 45). This approach gives an important role to animal health professionals and animal owners as well as to people in regular contact with domestic and wild fauna and the environment. To be effective, control actions must address all levels of society, all political, religious or associative groups. Although it primarily concerns the Ministries of Health and Agriculture or Livestock, rabies control also requires the involvement of the Ministries of the Interior, Education and Communication, and Research (36). All stakeholders, including universities, learned societies and associations of physicians, pharmacists and animal health professionals, all schools, the media, local authorities and religious groups, and even neighborhood communities, must be sensitized and involved in the fight against rabies if the disease is to be defeated in the near future.

## Conclusion

Socio-cultural and institutional factors influence a dogs' access to vaccination. Therefore, these factors, which may constitute barriers to rabies control, must be addressed and eliminated through context-specific communication strategies to improve vaccination and surveillance coverage. In Chad, this study demonstrated three major problems that impede dog vaccination. These obstacles are economic (cost of vaccines), sociocultural (belief of loss of valued characteristics of dog meat in case of vaccination on the one hand and stigmatization of dogs on the other), and institutional (unavailability of vaccine and lack of knowledge and communication of the law on dog vaccination).

To achieve efficient control and ultimately elimination of rabies the collaboration of several actors intervening in the field of human and animal health, safety, education, communication, research, environment, etc. is crucial. These actors need to have sufficient resources to respond to the existing demand for dog vaccine but also to engage in awareness raising to increase demand by dog owners.

## Data availability statement

The raw data supporting the conclusions of this article will be made available by the authors, without undue reservation.

## Ethics statement

The studies involving human participants were reviewed and approved by Ethics Committee of Northern and Central Switzerland (EKNZ). Written informed consent for participation was not required for this study in accordance with the national legislation and the institutional requirements.

## Author contributions

NM: sociologist, study design, data collection and analysis, and manuscript writing. AM: health geographer, study co-design, data collection, and analysis support. DA: sociologist, methodological, and analysis support. PN: veterinarian and manuscript writing support. JZ: veterinarian, one health expert, and overall study PI. KH-T: sociologist, methodological support, and manuscript revision. ML: veterinarian and rabies expert, study coordination, data collection, supervision, and manuscript revision. All authors contributed to the article and approved the submitted version.

## Funding

This research is part of a project conducted under the DELTAS Africa Initiative [Afrique One-ASPIRE /DEL-15-008] and the Global Vaccine Alliance (GAVI) learning agenda (Exhibit A-3 PP46311015A3C). Africa One-ASPIRE was funded by a consortium of donors including the African Academy of Sciences (AAS), the Alliance for Accelerated Scientific Excellence in Africa (AESA), the New Partnership for Africa's Development (NEPAD) Planning and Coordinating Agency, the Welcome Trust [107753/A/15/Z], and the UK government.

## Conflict of interest

The authors declare that the research was conducted in the absence of any commercial or financial relationships that could be construed as a potential conflict of interest.

## Publisher's note

All claims expressed in this article are solely those of the authors and do not necessarily represent those of their affiliated organizations, or those of the publisher, the editors and the reviewers. Any product that may be evaluated in this article, or claim that may be made by its manufacturer, is not guaranteed or endorsed by the publisher.
