# Supplementary material for: General insights on obstacles to dog vaccination in Chad on community and institutional level
Source: Front Vet Sci. 2022 Oct 12;9:866755. doi: 10.3389/fvets.2022.866755 (PMC9597194; doi:10.3389/fvets.2022.866755)
Supplement: Supplementary file 1 [file Data_Sheet_1.docx]

**Supplementary document Nr.1**

**Discussion points addressed during Focus Groups and interview questions by participant group**

**Points discussed during focus groups with dog owners**

1. How do you feel about the cost of rabies vaccination?
2. Is there a veterinary station in your community?
3. How far are you from the dog vaccination center?
4. How do you transport your dogs to the vaccination station?
5. What role do dogs play in your community?
6. How is the dog perceived in your community?
7. Do you appreciate the rabies vaccination of your dogs?
8. Are rabies vaccines available at the vaccination stations you frequent?
9. Is the cost of transportation affordable for you to bring your dogs to the vaccination?
10. What difficulties do you face in accessing vaccination for your dogs?
11. Are you aware of the dog vaccination law implemented by the authorities?
12. Your last word

**Points discussed during focus groups with veterinary officers and heads of livestock sectors:**

1. How do you define rabies?
2. Is rabies included in your training program?
3. What are the different activities that you conduct in the fight against rabies?
4. Do you have rabies vaccine available at your center?
5. Do you have a collaborative relationship with other health services?
6. What difficulties do you encounter in the fight against rabies?
7. Your final words

**Interview questions for dog owners:**

1. How do you feel about the cost of rabies vaccination?
2. How far are you from the dog vaccination center?
3. How do you transport your dogs to the vaccination station?
4. What do dogs represent in your community?
5. Do you value rabies vaccination for your dogs?
6. Are rabies vaccines available at the vaccination posts you frequent?
7. Does the cost of transportation allow you to bring your dogs to the vaccination?
8. What difficulties do you face in accessing vaccination for your dogs?
9. Are you aware of the dog vaccination law implemented by the authorities?
10. Your last word

**Interview questions for civil society organizations:**

1. What does the dog represent in your community?

2. What do you think rabies is?

3. How can rabies be controlled?

4. What factors hinder access to dog vaccination in your community?

5. What are the consequences of not vaccinating dogs?

6. What do you think should be done to increase access to dog vaccination?

**Interview questions for delegates of veterinary districts and chief medical officers:**

1. How can rabies be controlled?

2. Is there a police health law for dog vaccination?

3. How is this law popularized?

4. Does the population respect this law?

5. What prevents the application of this law?

6. What do you think are the difficulties related to access to dog vaccination?

7. What actions do you think should be taken to allow access to dog vaccination?

**Interview questions for veterinary officers and heads of livestock sectors:**

1. How do you define rabies?
2. Is rabies included in your training program?
3. What are the different activities that you conduct in the fight against rabies?
4. Do you have rabies vaccine available at your center?
5. Do you have a collaborative relationship with other health services?
6. What difficulties do you encounter in the fight against rabies?
7. Your final words
